# Regulation of nitrogen dynamics at the sediment–water interface during HAB degradation and subsequent reoccurrence

**DOI:** 10.1039/c9ra10673a

**Published:** 2020-04-04

**Authors:** Weiping Sima, Meijuan Hu, Qiang He, Yixi Qiu, Yitao Lv, Lichun Dai, Qingwei Shao, Tao Zhou, Hong Li, Manyu Zhou, Hainan Ai, Hao Zhan

**Affiliations:** Department of Civil Engineering, Sichuan University of Science and Engineering Zigong 400045 P. R. China aihainan@126.com zhanhao1995@foxmail.com; Key Laboratory of Eco-Environment of Three Gorges Region, Ministry of Education, Chongqing University Chongqing 400044 P. R. China; Biomass Energy Technology Research Center, Biogas Institute of Ministry of Agriculture Chengdu 610041 P. R. China; The Tibet Autonomous Region Housing and Urban-rural Development Hall Job Training Center China; Chongqing Haoyang Water Construction Management Co., Ltd. Chongqing 400020 PR China

## Abstract

The effects of harmful algal blooms (HABs) on nutrient dynamics have been extensively studied; however, the response of nitrogen to continuous HAB degradation and subsequent reoccurrence is not well understood. Here, a small-scale experiment was conducted to assess how nitrogen in the sediment–water interface (SWI) responds to HAB degradation and subsequent reoccurrence at different initial algal densities. The results showed that during the algae decomposition stage, the NH_4_^+^–N flux of the SWI remained positive but decreased with the increase in algal density from 3.5 × 10^7^ to 2.3 × 10^8^ cells per L, indicating that the sediment was the source of NH_4_^+^–N. In contrast, the deposit was a sink of NO_3_^−^–N. However, during the reoccurrence of HAB, the distribution of NH_4_^+^–N and NO_3_^−^–N fluxes was completely converted. Nitrogen flux analysis throughout algae decomposition and reoccurrence indicated that although the sediment acted as a sink of nitrogen, the flux was dependent on the initial algal density. Our results confirmed that algae decomposition and reoccurrence would greatly affect the nitrogen cycle of the SWI, during which dissolved oxygen (DO) and initial algal density dominated. This study is the first to show that the regulation of nitrogen flux and migration changes during continuous HAB decomposition and subsequent reoccurrence.

## Abstract 2

The effects of harmful algal blooms (HABs) on nutrient dynamics have been extensively studied; however, the response of nitrogen to continuous HAB degradation and subsequent reoccurrence is not well understood.

## Graphical abstract

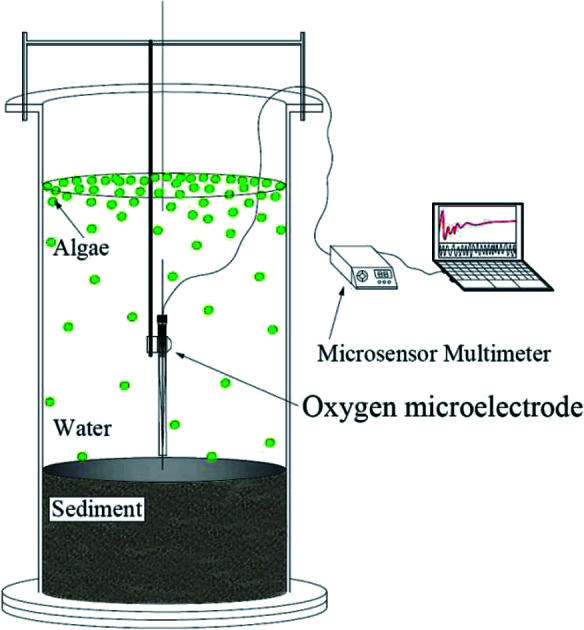

## 1. Introduction

As a global environmental problem, eutrophication, which is responsible for harmful algal blooms (HABs), has attracted increasing attention.^1^ Severe HABs can cause a variety of ecological and environmental problems, including serious threats to biodiversity, resulting in hypoxia during the decomposition phase and the release of toxic metabolites.^2,3^

Previous studies on HAB formation have found that nitrogen (N) and phosphorus (P) can stimulate the growth of phytoplankton, so excessive inputs of N and P promote excessive nutrients and HABs.^4^ An experimental study evaluated 143 lakes on the latitudinal cross-section from the sub-Arctic to southern South America and found that temperature and TN concentrations were key factors influencing cyanobacterial biomass.^5^ Other studies have also found that the presence of N increases the microcystin quota of harmful *Microcystis aeruginosa*.^6^

However, aquatic ecosystems do not always respond passively to HAB. Recent studies found an important link between denitrification in cyanobacteria and eutrophic lakes.^7^ When the maximum bloom developed, 40% of the lake area was covered with dense patches of the bloom, and a large amount of nitrogen was released when the algae bloom decayed in Lake Atitlan.^8^ A recent study found that under the pressure of limited nitrogen availability, the induction of organic matter and carbohydrate accumulation and a high C/N ratio in cyanobacteria could be observed, potentially serving as a promising carbon source for denitrification.^9^ Lei *et al.* studied the feasibility of planting algae while removing nutrient and biomass production.^10^ Furthermore, contrary to the nutrients of active phytoplankton species, the decomposition of algae cells can release large amounts of inorganic and organic nutrients that constitute an important pool of nutrients in the water and ultimately become a source of endogenous contamination of sediments.^11^

The effects of algae decomposition on nutrient dynamics have been extensively studied. Algae decomposition and subsequent recovery play an integral role in nutrient transfer and transformation. However, little research has been conducted on how algal decomposition and recovery processes affect nitrogen dynamics. The effects of phytoplankton reproduction and decomposition may be the most important factors. However, the effects of phytoplankton decomposition and recovery on the water environment and the nitrogen dynamics of SWI are still unclear.

SWI is the transition zone between water and sedimentary facies in an aqueous environment and plays an important role in the circulation, transfer and storage of matter in the water environment.^12^ Nitrogen conversion in SWI is affected by concentration gradients, dissolved oxygen and pH.^13^ However, this process could be greatly impacted by phytoplankton. When several HABs occur and dense algal cells cover the water surface, the water column becomes oxygen-depleted, and the organic matter content in the water increases. When HAB is not as severe as the scenario described above, the decomposition process of algae multiplication may be accompanied by resuscitation, resulting in absorption and release of nutrients. As long as the temperature and light are appropriate, the algae will continue to be active and perform photosynthesis, resulting in changes to the oxygen flux in the SWI, which affect the migration and transformation of nitrogen throughout the water. There are few reports on whether this process promotes or inhibits the release of endogenous nitrogen in the sediment. Furthermore, it is unclear how the decomposition and recovery of HABs affect the nitrogen cycle of SWI.

Here, a small-scale experiment was conducted to assess the decomposition and subsequent recovery of cyanobacteria-led HAB. We explored the nitrogen dynamics of the SWI and the underlying mechanisms at different initial cyanobacterial densities. The purpose of this study was to elucidate the role of cyanobacteria in the dynamics of nitrogen in SWI. This result will help to elucidate the contribution of HAB to nitrogen dynamics throughout the degradation and recovery phase.

For this study, we propose three necessary hypotheses for the study: (1) algae decay and recovery dominate dissolved oxygen and microbial nitrogen dynamics in the sediments; (2) no algae cells in the sediment; (3) the effect of nitrogen input in the gas phase on the sediment–water system is not considered.

## 2. Materials and methods

### 2.1. Incubation experiment

In this experiment, incubators made of plexiglass cylinders were used to study the effects of phytoplankton decomposition and recovery on nitrogen dynamics. The incubator has an inner diameter of 20 cm and a height of 80 cm. Each of the incubators was filled with 25 cm of thick sediment, which was sieved to remove rocks and plant residues. The sediment originated from Minzhu lake, a small contaminated lake in China, which was affected by domestic sewage and rainwater runoff, showing a state of eutrophication all year round, and associated algal blooms. The amount of water in each reactor was brought to 16 L. The water was filtered by a 0.22 μm membrane to remove phytoplankton. Then, 18.7 mL, 29.3 mL, 80 mL, 101.3 mL and 122.7 mL of condensed algae (3.0 × 10^10^ cells per L) collected from Dianchi were added into reactors. As a result, the initial densities of these 5 systems were 3.5 × 10^7^ cells per L, 5.5 × 10^7^ cells per L, 1.5 × 10^8^ cells per L, 1.9 × 10^8^ cells per L, and 2.3 × 10^8^ cells per L, respectively. Phytoplankton was not added to the control group, and all experimental groups were conducted in triplicate. All incubators were held in the dark for 6 days^14,15^ to attempt to induce algal cell decomposition. The dark treatment is only to induce the phytoplankton to decline, and the continuous 12 h light and 12 h dark cycle for 11 days is a simulated natural environment. Continuous monitoring of data during these 11 days and the dissolved oxygen concentration in Fig. 3 is the data measured with a DO microelectrode at the same water depth for each reactor from 9 to 10 am every day, and the average value of dissolved oxygen within 1 minute is taken. So, in fact, we only measured the dissolved oxygen value under light condition. Then all incubators were incubated under an alternating cycle of 12 h of light and 12 h of darkness at 25 °C or 11 days. The initial concentrations of NH_4_^+^–N and NO_3_^−^–N were 4.79 mg L^−1^ and 0.68 mg L^−1^, respectively, in the pore water of the sediment and 1.60 mg L^−1^ and 0.73 mg L^−1^, respectively, in the water column. All indicators were analysed by standard methods.^16^

### 2.2. Sample collection and preparation

The algal cells were enumerated in a counting chamber of an electromotive microscope (Axioskop 2 mot plus, Carl ZEISS, Germany) after fixation in Lugol solution.^17^ The water samples (10 mL in each incubator) were collected at 5 cm below the water surface using a pipette. The sediment samples (20 mL in each incubator) were collected at 10 cm below the SWI using a syringe, then centrifuged at 4000 rpm for 10 min to obtain the pore water. Chla samples were collected with 0.22 μm membrane filters and extracted by acetone (90%) for 24 h at 4. Ammonium (NH_4_^+^–N) and nitrate (NO_3_^−^–N) contents of the overlying and pore water were measured by Nessler's colorimetric method and an ultraviolet colorimetric method, respectively. The *F*_v_/*F*_m_ index (characterization of the vitality of algal cells) of the algae in each device was measured by an AquaPen-C 100.^18^*F*_v_/*F*_m_ represents the maximum photochemical efficiency of Photosystem II (PSII) under dark adaptation, also known as the energy capture efficiency of the open PSII reaction center. Where *F*_v_ (*F*_v_ = *F*_m_ − *F*_o_) represents the maximum variable fluorescence; *F*_o_ represents minimum fluorescence (or basic fluorescence, *etc.*), which is the fluorescence intensity when all PSII centers of fully dark-adapted photosynthetic apparatus are open; *F*_m_ represents maximum fluorescence, which is the fluorescence intensity when all PSII centers are closed for fully dark-adapted photosynthetic conditions. The oxygen flux in the SWI during the illumination treatment was measured using a DO microelectrode testing system at the same water depth for each reactor from 9 to 10 am every day under light condition, and the average value of dissolved oxygen within 1 minute is taken. The water quality and algal *F*_v_/*F*_m_ were consecutively measured for 11 days, and samples were taken every 24 h.

### 2.3. Calculation of the nitrogen and oxygen flux in sediment–water interfaces

The NH_4_^+^–N and NO_3_^−^–N flux in SWI were calculated according to the following equation:^19,20^
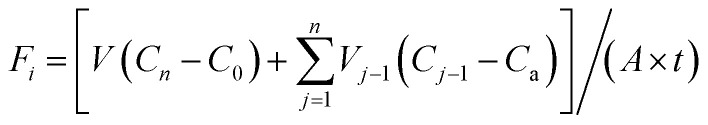
where *F*_*i*_ represents the average flux of NH_4_^+^–N or NO_3_^−^–N until day *i* (mg m^−2^ d^−1^); *V* represents the volume of overlying water in the experimental column (L); *V*_*j*−1_ is the volume of water samples collected on day *j* − 1 (L); *C*_0_, *C*_*n*_ and *C*_*j*−1_ represent the concentration of NH_4_^+^–N or NO_3_^−^–N on day 1, day *n* and day *j* − 1 of sampling (mg L^−1^), respectively; *C*_a_ is the concentration of NH_4_^+^–N or NO_3_^−^–N in the added-water sample (here, *C*_a_ is zero due to the addition of ultra-pure water); *A* is the area of the SWI (m^2^); and *t* represents the interval time of sampling (day). All nutrient fluxes in this paper were the cumulative results for 11 days.

### 2.4. Statistical analysis

**Fig. 1:**
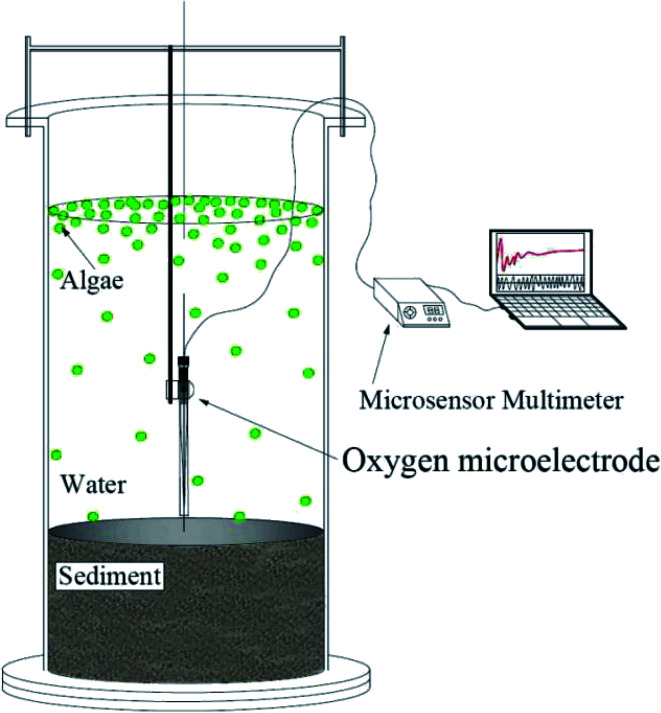
Schematic diagram of the experimental system.

Graphs were plotted using Origin 8.5 (Origin Lab, Northampton, MA, USA). The *t-*test was used to determine significant differences among different measurements. Significance was assigned when *P* < 0.05 (Fig. 1).

## 3. Results and discussion

### 3.1. Alterations of algal density and photochemical efficiency

**Fig. 2:**
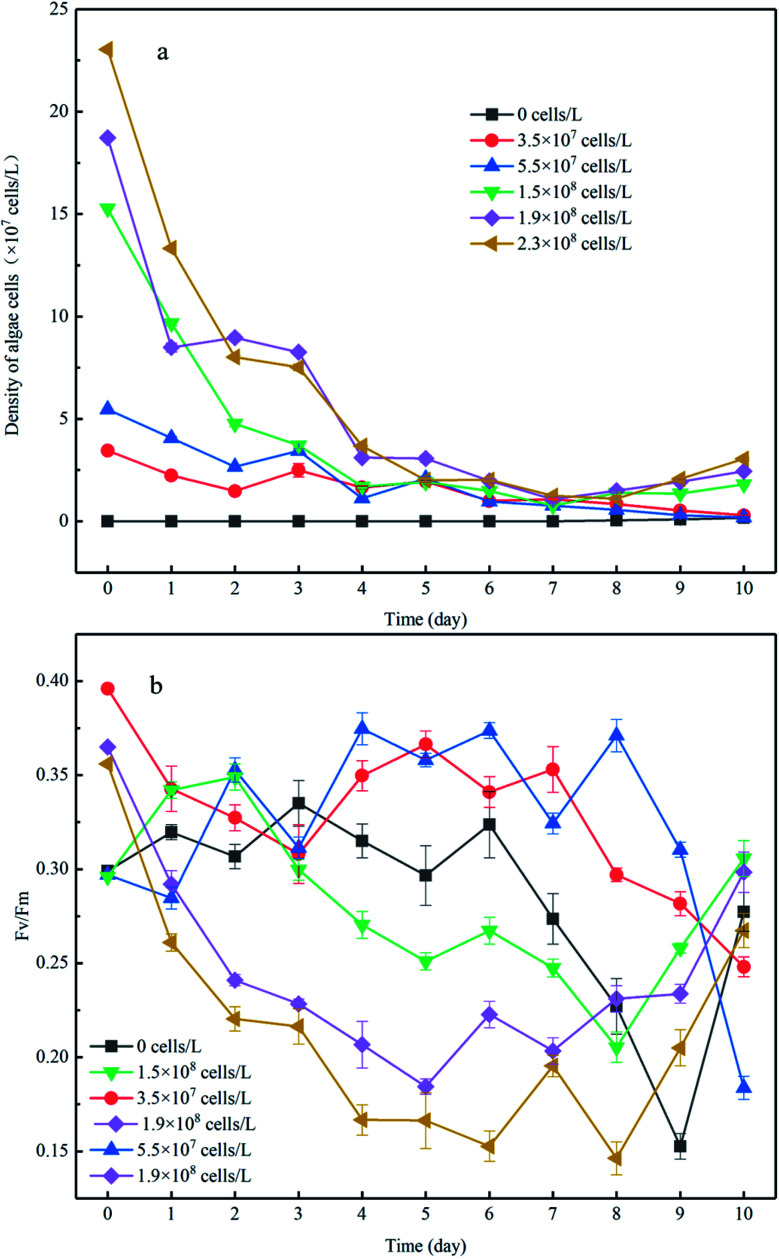
The changes in algae cell density in the overlying water (a) and *F*_v_/*F*_m_ of algae cells (b), with an initial density of algae cells to distinguish each experimental group. The data are the mean value of triplicates. Error bars denote ±1 standard error.

There was no increase in algal cell density in the control group throughout the experiment; however a sudden decrease in algae density was observed in the experimental group to which algae was added (Fig. 2a). After 8 days of incubation, the cell density of the high-algal-density group (algal density > 10^8^ cells per L) began to increase. Consequently, we theoretically divided the experimental process into two phases: in phase I (from day 1 to day 8), the algae degraded and settled into the sediment, and in phase II (from day 9 to day 11), the algae cells recovered. In phase I, the algal cell density in the high-density group decreased from 2.3 × 10^8^ cells per L to 2.8 × 10^7^ cells per L, and the reduction rate was 9 times higher than that in the low-density group (Fig. 2a). It should be noted that nutrient limits, temperature, exposure to light and toxic substances can cause cell death when the phytoplankton biomass reaches its peak.^21^ In this experiment, the number of phytoplankton cells differed by an order of magnitude and thus showed different growth and decline trends. Previous studies have shown that phytoplankton communities coexist in shallow lakes while they compete for nutrients (N and P) and light.^22^

*F*_v_/*F*_m_ is closely related to the density of algae and can be used to characterize the photochemical efficiency of cyanobacteria cells, that is, the activity of algae cells. The larger the *F*_v_/*F*_m_ value, the higher the photochemical efficiency of the cyanobacteria cells and the stronger the activity of the algae cells. The value of the low-density group in phase I was in the range of 0.3–0.4 (Fig. 2b), which may be the reason for the low rate of algae density decline. When the initial algal density was approximately 10 times higher, the minimum value of *F*_v_/*F*_m_ showed a nearly 37% reduction. This is because the fewer algal cells in the system, and the more nutrients and light available, the more active the algal cells. This is also supported by previous studies that show phytoplankton communities co-existing in shallow lakes and competing for nutrients (N and P) and light.^23^ In addition, for the experimental group with low algal density, the algal cell density declined slowly despite the relatively strong activity of algal cells, indicating that some algal cells with strong activity continued to grow and reproduce in the microcosmic system at this time, but the number of decay and sedimentation of algal cells was greater than the amount of proliferation. On the contrary, both the cell density and *F*_v_/*F*_m_ value of the experimental group with high algal density decreased rapidly, indicating that the decline and death of cyanobacteria bloom was dominant in the system. This was because the higher the algal density was, the more competition algal cells had for nutrients and living space, which would further hinder the growth and proliferation of algal cells. This is consistent with the findings of some scholars., that when phytoplankton biomass reaches its peak (algal density > 10^8^ cells per L), the resulting nutritional stress and exposure to extreme light, temperature and toxic substances may lead to cell death.^24^ Moreover, during phase I, when the initial algal densities were 3.5 × 10^7^ cells per L and 5.5 × 10^7^ cells per L, the *F*_v_/*F*_m_ showed slight variation despite a dramatic decrease that occurred until day 7, in comparison to the constant reduction of *F*_v_/*F*_m_ when the initial algal cell density increased. This was supported by a previous study showing that the competition for nutrients and living space could be enhanced when the algal density increased,^25^ which would further impair the photochemical efficiency.

During phase II, the *F*_v_/*F*_m_ of the low-density group was further reduced to 0.1 and 0.12, respectively, and did not return to its previous level until the end of incubation. Considering the profile of algal density (Fig. 2a), we conclude the algal cells had almost decomposed in the low-algal-density groups, which was consistent with a previous study.^26^ In contrast, the photochemical efficiency of the high-density group continuously increased and almost returned to the initial activity from day 8 to day 11 (Fig. 2b), indicating that algal blooms were accompanied by recovery after decomposition of the algae. The reasons of this phenomenon may be: (1) phase I the decline and fall of algal cells in promoting the transformation of the migration of nutrients in micro cosmic system, increase the nutrient of overlying water algae cells required; (2) during the decay process, the *F*_v_/*F*_m_ value of algal cells did not drop to zero, indicating that there were still very few algal cells with strong activity. (3) temperature, light and other external conditions are suitable for algal cell growth and reproduction.

### 3.2. DO profile in the overlying water

**Fig. 3:**
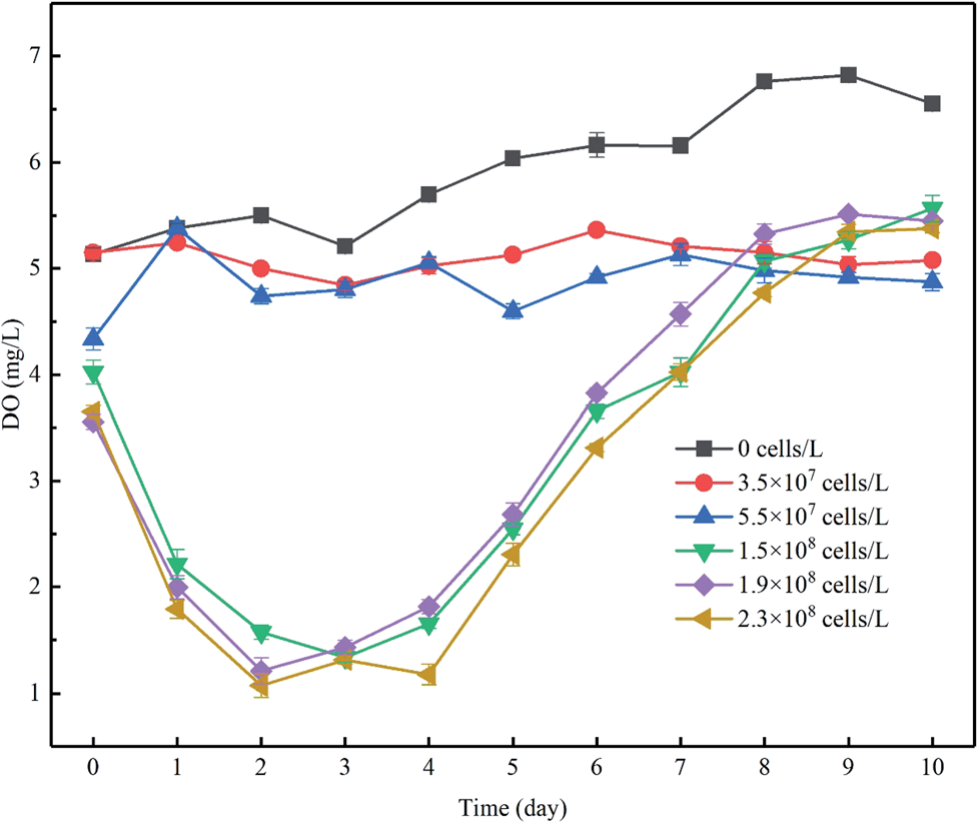
The changes in DO concentration in the overlying water (mg L^−1^). DO concentrations in each experimental group were assessed, and the most gentle 1 minute DO concentration (calculated the average (*C̄*)) data from the real-time DO concentrations are shown. Error bars denote ±1 standard error.

The increase of algal density and photochemical efficiency would promote the photosynthesis of algae cells to produce oxygen,^27^ changing the concentration gradients of oxygen in the overlying water as a result, and DO is a key control factor for microbial participation in the nitrogen cycle.^28^ During the experiment, the concentration of DO in the control group ranged from approximately 5.2 to 7 mg L^−1^ and was significantly higher than that in the experimental group to which algae was added, but lower than the corresponding water dissolved oxygen (8.06–8.14 mg L^−1^) under indoor temperature and atmospheric pressure. The lower DO was probably caused by the consumption of DO in the water by organic matter and microorganisms in the sediments. In phase I, when the algae density was kept at a low level, the DO concentration increased by 7.9 to 17.7%. When the initial cell density increased, the DO level increased by 28.3 to 49.3% despite the decrease in density (Fig. 3). Although the DO increase rate in the high-algal-density groups far exceeded that in the lower-density groups, the DO concentration did not follow the same pattern, which may be attributable to the following reasons: (1) more nutrients, particularly algae-originated organic matter, could be released from the decay of algae cells in the groups with high algae density, leading to the consumption of DO in the overlying water,^13^ and (2) considering the limited decreasing rate of algal density in the groups with low algal density (Fig. 2a), and despite the proliferation of active algae cells and high oxygen production of photosynthesis, the algae with weak vitality decayed, and the rate of algal cell proliferation was lower than its decomposition, which would in turn consume DO. Furthermore, this was confirmed by the fact that the overall density of algal cells decreased (Fig. 2a), but the *F*_v_/*F*_m_ was relatively high in the low-algal-density groups (Fig. 2b).

During phase II, the resurgent algae cells increased the intensity of photosynthesis. As a consequence, the DO concentration increased in each group. After the end of 11 days of incubation, the DO concentration in the overlying water in each treatment (groups added with algae except the control group) further increased, although the rates of increase were different (Fig. 3).

### 3.3. Close relationship among NH_4_^+^–N, NO_3_^−^–N flux and initial algal concentration during decomposition and recovery

**Fig. 4:**
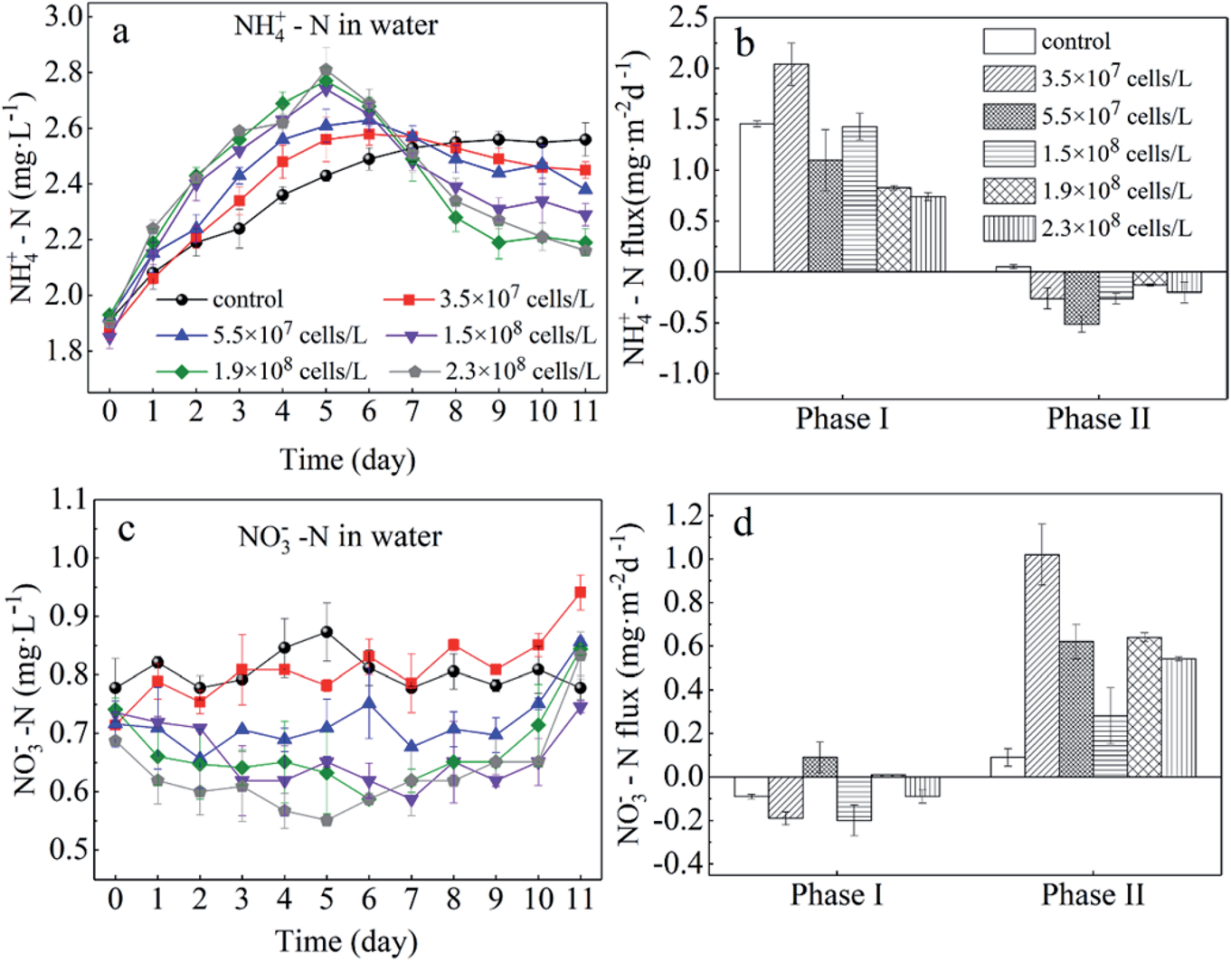
Sediment to water flux of NH_4_^+^–N (b) and NO_3_^−^–N (d) and concentrations of NH_4_^+^–N (a) and NO_3_^−^–N (c) in the overlying water. Error bars denote ±1 standard error.

**Fig. 5:**
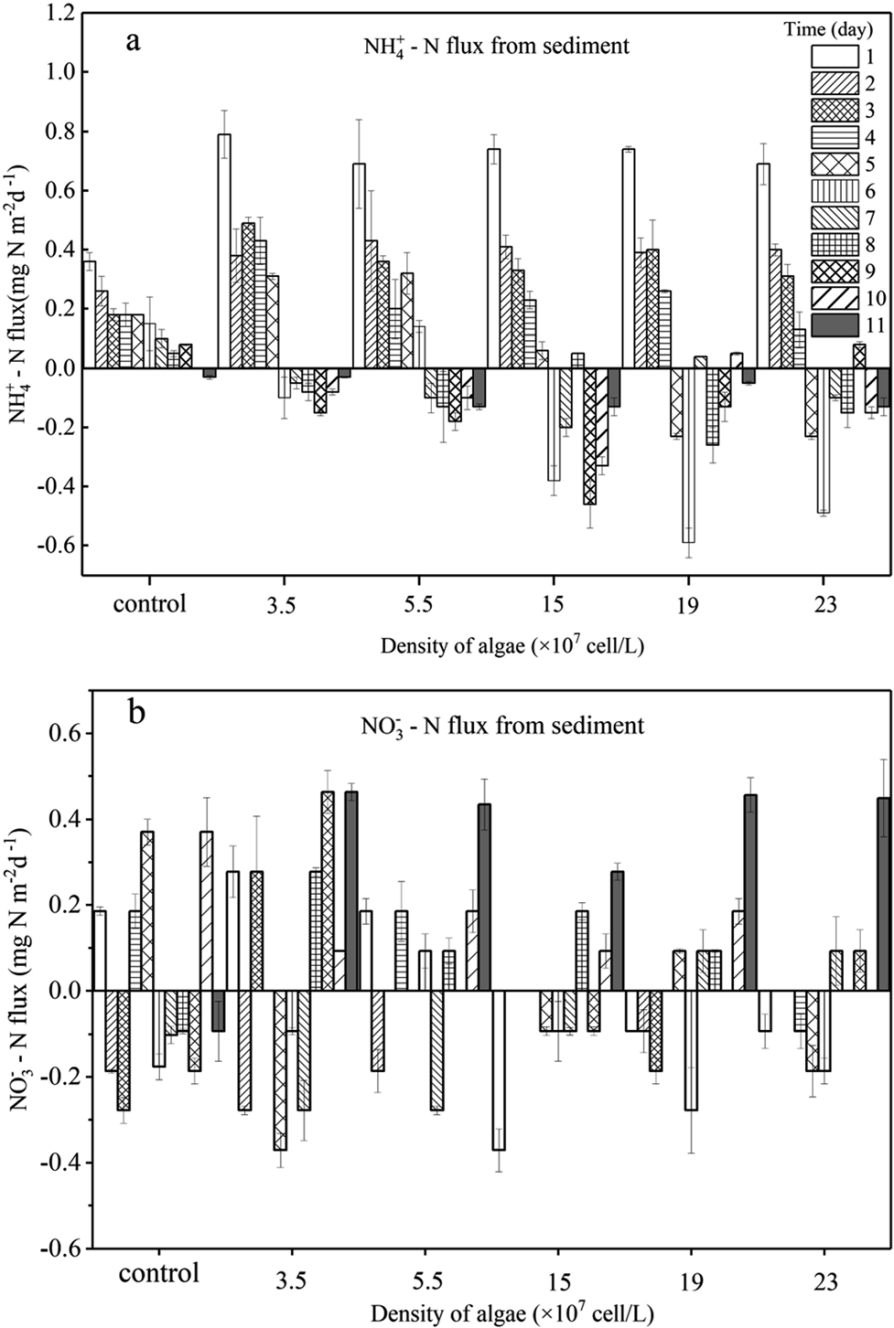
Sediment to water flux of NH_4_^+^ –N (a) and NO_3_^−^–N (b) daily. Error bars denote ±1 standard error.

In the present study, we evaluated the different roles of phytoplankton in the two phases of nitrogen migration. According to Fig. 4 and 5, it was obvious that in phase I, the variation of the NH_4_^+^–N concentration had its own regularity compared with the NO_3_^−^–N concentration, which tended to change in a disorderly manner. However, in phase II, the NO_3_^−^–N concentrations of several treatments increased to different degrees, indicating that during the decomposition of algae, because of the limitation of DO, nitrification was not the main reaction, while during the recovery afterward, the intensity of nitrification started to grow; it appeared that algal density influenced the intensity of nitrification, but instead it was the DO concentration that had an effect.

During phase I, when algae cells were absent (control), the NH_4_^+^–N concentration in the overlying water increased slightly from 1.91 mg L^−1^ to 2.55 mg L^−1^ (Fig. 4a) and stabilized starting at day 9. Subsequently, the NH_4_^+^–N concentration in the aqueous phase reached 2.60 mg L^−1^, indicating that sediment was the inner source for the NH_4_^+^–N of the overlying water. Furthermore, during this process, the driving force of NH_4_^+^–N release from sediment in the control group was derived from the concentration gradient. The NH_4_^+^–N concentrations in the overlying water of the five algae-incubated microcosms increased significantly, which was also observed in the control group, and the initial density of algae was positively related to the variation of the NH_4_^+^–N concentration. The difference in the concentration of NH_4_^+^–N in the overlying water between the treatment groups can be attributed to the difference in the initial density of the added algae and the number of dead algal cells. This result showed that the speed of decomposition was fast when the algae was in a decline phase, and approximately 60% of the algae could be decomposed in 5 days,^29^ leading to a sharp increase in the concentration of nutrients in the water system. This also indicated that the decomposition of declining algae could raise the concentration of NH_4_^+^–N rapidly.^11,30,31^ Moreover, the diffusion of NH_4_^+^–N from sediment and the function of microorganisms could also contribute to the variation of NH_4_^+^–N in the overlying water. However, this effect was eliminated by the control group. As a result, among the 5 microcosm groups with algae, the high initial density of algae led to a high NH_4_^+^–N concentration in the overlying water.

For the groups with low algal density, the *F*_v_/*F*_m_ value and DO concentration both decreased a little but were still higher than those in the high-algal-density groups. In addition, the decreasing rates of algae density were low, showing that phytoplankton did not completely decline, and a few active algae cells continued to grow and reproduce. Hence, the reasons for the variations of NH_4_^+^–N caused by the activity of algae in the microcosm were as follows: (1) a small number of algae decomposed and released NH_4_^+^–N; (2) the algae conversely consumed the NH_4_^+^–N during the processes of growth and reproduction,^32^ as when nitrogen nutrients are sufficient, NH_4_^+^–N is the prior nutrient utilized by algae;^33^ (3) since the number of nitrifying bacteria in the water body is much less than the sediment,^34^ the possibility of consuming NH_4_^+^–N in the overlying water through nitrification is also very limited;^35^ (4) the possibility of anaerobic ammonia oxidation in organic-rich eutrophic lakes is also very small.^36^ Given that the number of nitrifying bacteria in a water body is far less than that in the sediment,^37^ and anaerobic ammonium oxidation is unlikely to occur in eutrophic lakes with abundant organics,^38^ in the groups with low algal density, the assimilation of NH_4_^+^–N in the overlying water led the concentration gradient between the sedimentary phase and aqueous phase to increase, driving NH_4_^+^–N to diffuse to the overlying water, which can also be illustrated by the NH_4_^+^–N flux (Fig. 5a). In the first 5 days, the NH_4_^+^–N fluxes in the low-algal-density groups were larger than those in the high-algal-density groups. Furthermore, their NO_3_^−^–N fluctuated a little in phase I, which could be attributed to the absorption of sediment (Fig. 4d), assimilation by algae and nitrification.

With respect to the high algal density groups, in the first five days of phase I, the algal density (Fig. 2a) and *F*_v_/*F*_m_ (Fig. 2b) were significantly reduced. The DO concentration dropped dramatically (Fig. 3), indicating that the phytoplankton in the microcosm was in the decline phase, and the increase of NH_4_^+^–N in the overlying water was mainly attributed to the release from algal cells, which led to the higher NH_4_^+^–N concentration gradient. As the algae collapsed, the NH_4_^+^–N concentration gradient between the sedimentary phase and aqueous phase fell rapidly. Additionally, when the NH_4_^+^–N concentration in the overlying water was higher than that in the sediment, a reverse NH_4_^+^–N flux was observed at day 5, and the higher the algal density was, the earlier this reverse occurred. After 5 days, the decay rate of algae in the high algal density test group was close to that in the low algal density test group, indicating that the role of NH_4_^+^–N released by algal cell decay was weakened. At this time, the concentration of NH_4_^+^–N in the overlying water begins to decrease. Due to the difference in concentration, a small amount of NH_4_^+^–N may diffuse into the sediments, and the rest may go to assimilation, nitrification or anaerobic ammonia oxidation. Considering 5–8 days algal density continues to slow down and *F*_v_/*F*_m_ value is still in the lowest knowable assimilation almost does not exist, and anaerobic ammonia oxidation occurred in the eutrophication of lakes that are rich in organic matter may also be tiny,^36^ that is probably the nitrification process in overlying water continued consumption of NH_4_^+^–N, and the high concentration of NH_4_^+^–N and overlying water were elevated the DO concentration also has the nitration reaction conditions.^34,39–43^ Therefore, the concentration of NH_4_^+^–N in the overlying water of the high algal density microcosm system began to decline after reaching the highest value on the 5th day (Fig. 4a).

In brief, a positive flux of NH_4_^+^–N from sediment to the overlying water was observed in controls (1.49 mg m^−2^ d^−1^) and algae-added systems (ranging from 0.45 to 1.46 mg m^−2^ d^−1^, except for the algal density of 3.5 × 10^7^ cells per L), and it was apparent that the NH_4_^+^–N flux showed a negative relationship with the initial algal density (Fig. 4b). This illustrated that low algal density could boost NH_4_^+^–N diffusion from the sediment to the overlying water. On the other hand, high-algal-density groups inhibited the release of NH_4_^+^–N from sediment during the decline phase.

After the collapse of severe HABs (generally, the algal cell density reached 10^9^ cells per L or higher), some algae recovered and continued to proliferate.^44^ Similar observations were also reported by several recent studies.^45,46^ However, there has been little discussion about the performance of nitrogen in the recovery process. Overall, during recovery (phase II), the NH_4_^+^–N in the overlying water of low-algal-density groups varied little, and the concentration of NH_4_^+^–N was maintained around the peak value. However, it dropped dramatically in the high-algal-density groups. Furthermore, higher algal densities dropped more rapidly. However, NH_4_^+^–N fluxes in the systems with algae all reversed to negative values, although the total flux was small, and the fluxes of low-algal-density groups were higher than those in high-algal-density groups. In the end, whether NH_4_^+^–N transfers from sediment to water or not depends on the initial algal density.

The *F*_v_/*F*_m_ values in the groups with low algal density continued to decrease from 0.3 to 0.1 or even less during phase II (Fig. 2b), indicating that the algal cells barely recovered. Additionally, because of reoxygenation from the atmosphere, the DO concentration in the overlying water rose to 5.0 mg L^−1^ (Fig. 3), which was beneficial for nitrification in the surface of sediment, and consequently NO_3_^−^–N was released into the overlying water, leading to the increase of NO_3_^−^–N in phase II (Fig. 4c). At the same time, nitrification resulted in the increase of NH_4_^+^–N concentration difference between sediment and water phase, thus promoting the diffusion of NH_4_^+^–N in the overlying water to the sediment. As a result, 3.3–4.2% of NH_4_^+^–N decreased in the overlying water (Fig. 4a), and the rate of diffusion remained low (Fig. 5a). In phase II, *F*_v_/*F*_m_ increased from 0.15 to 0.37 in the groups with high algal density (Fig. 2b), and furthermore, the DO concentration in the aquatic phase rose to 5 mg L^−1^ (Fig. 3). The rate of NH_4_^+^–N decrease in the aquatic phase with higher algal density was high, which was not only due to the nitrifying process but also to absorption by the recovery of algae.^47^ It has been affirmed that when NH_4_^+^–N and NO_3_^−^–N both exist in a system, blue-green algae tend to use NH_4_^+^–N first;^33^ therefore, NO_3_^−^–N can accumulate in the overlying water (Fig. 4c). According to Fig. 3, the DO concentrations of 5 treatments with the addition of algae reached similar levels, and as a result, their NO_3_^−^–N fluxes resembled those at the end of experiment, indicating that it is the DO concentration rather than the initial algal density that plays the key role in the variation of NO_3_^−^–N. This is because the DO concentration of overlying water in all experimental groups at this time was almost greater than 5 mg L^−1^ (Fig. 3), which put the sediments in an aerobic environment and accelerated the aerobic nitrification process in the sediments.^13,34,39–43^ Moreover, the surface sediment is the main nitrification site, because aerobic nitrification bacteria are mostly about 1 mm in the surface of the sediment.^42^ Nitrification increased the concentration difference of NH_4_^+^–N between the sediment and water phase, resulting in the diffusion of NH_4_^+^–N from the overlying water into the sediments. Although NH_4_^+^–N was absorbed by the resuscitation of algal cells in the overlying water of the high algal density test group, the amount of increased algal cell density showed that NH_4_^+^–N was limited for cell assimilation of the resuscitating algal cells (Fig. 2a), and the main removal of NH_4_^+^–N in the overlying water was for nitrification. In addition, since resuscitated cyanobacteria cells photosynthesize to produce oxygen,^48^ more DO can be promoted to diffuse into the sediments, further intensifying the nitrification process on the surface of the sediments. As a result, the high density of algae in the experimental group, NH_4_^+^–N in the sediments and overlying water is consumed at the same time, but the consumption rate of sediment is bigger, lead to NH_4_^+^–N flux is negative, and than the low density of algae in the experimental group NH_4_^+^–N the absolute value of the flux is lower, because the low density of algal group in the late algal cell density did not increase, there is no recovery assimilation, algal cell group reduce NH_4_^+^–N used in surface sediments of nitrification. Consequently, the concentration difference dropped across the SWI, and the fluxes of high-algal-density groups were smaller than those in low-algal-density groups during phase II.

Although only 4 days of recovery were observed in the high-algal-density groups, according to algal density and increasing *F*_v_/*F*_m_ value, recovered algae was likely to continue to grow and even reach the algae bloom density a second time. Despite the lack of input of other nutrients, quite a large number of blue-green algae and bacteria possess the ability to fix nitrogen,^27,49^ and NH_4_^+^–N fluxes can reverse to be positive again.

### 3.4. Environmental implication

**Fig. 6:**
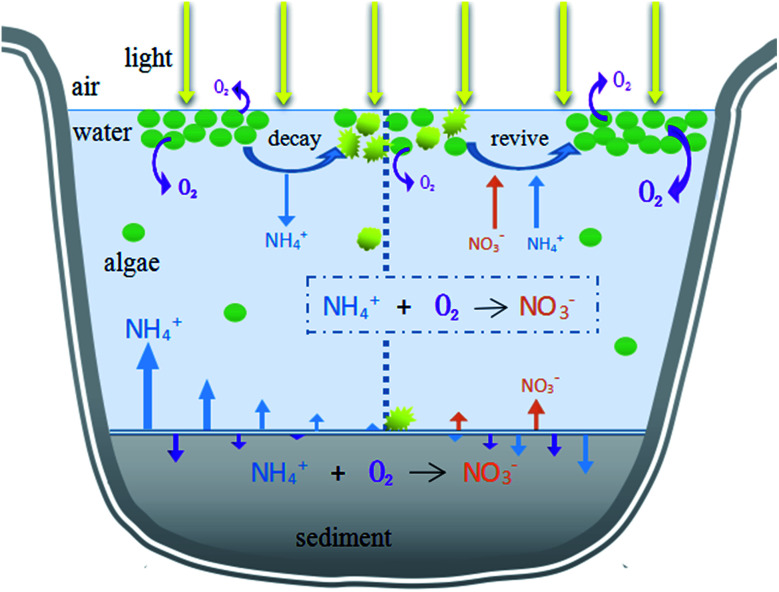
Illustration of the multiple approaches involved.

This research aimed to study the impact of the decline of HABs on migration and transformation of nitrogen in the system under different initial algae densities, in order to propose theoretical evidence to manage HABs and control nitrogen nutrients. During the different stages of algae decay and recovery, the nitrogen morphology was mainly nitrate or ammonia nitrogen. In the past, algal blooms were generally treated by mechanical salvage or overlaying precipitation. However, algal blooms do not only negatively affect aquatic ecosystems, but also may have a reaction mechanism on nutrients in aquatic ecosystems. Our study found that in different stages of algal bloom decay and recovery and under different algal densities, the main forms of nitrogen in hydrofacies and sediments were different, which provided scientific theoretical basis for formulating effective and feasible algal bloom treatment methods. The initial algal density reached 10^7^ cells per L, exceeding the critical value (10^6^ cells per L) of HABs. According to the results, in order to manage HABs in eutrophic lakes and control nitrogen at different algal flowering levels, it is a priority to identify the algae level and stage. Different actions should be taken according to different degrees of algal bloom. If the density of algae is low (<10^8^ cells per L), movements such as salvage to control the HABs should be taken before the algal density drops rapidly because of the assimilation of NH_4_^+^–N by algae, which could also eliminate nitrogen in the form of NH_4_^+^–N from a water system. For HABs with high algal density (>10^8^ cells per L), actions should be taken in the later decline phase to transfer the NH_4_^+^–N to NO_3_^−^–N, reducing the concentration of NH_4_^+^–N. Furthermore, the increased NO_3_^−^–N can be eliminated by denitrification through the addition of extra bacteria, man-made environmental changes and the sediment reaction itself. In addition, according to the water environment effects, decisions about which phase to deal with HABs depend on how many main contaminants exist in the system, and specific advice on choosing the right intervention method is illustrated in Fig. 6.

## 4. Conclusions

Based on the present study, dual roles of HABs in nitrogen dynamics were observed. The decomposition of algal cells led to the positive flux of NH_4_^+^–N. In comparison, during the recovery of algal activity, the positive NO_3_^−^–N flux at the SWI was induced despite the appearance of a negative NH_4_^+^–N flux. Our findings indicated that multiple approaches were involved during the alternation of nitrogen dynamics, including algae adsorption, decrease or increase in NH_4_^+^–N diffusion, and migration of NH_4_^+^–N to NO_3_^−^–N through nitrification, during which the initial algae density and DO level played vital roles.

## Conflicts of interest

The authors declare no competing financial interests.

## Supplementary Material
