# Correction: Regulation of nitrogen dynamics at the sediment–water interface during HAB degradation and subsequent reoccurrence

**DOI:** 10.1039/d0ra90050h

**Published:** 2020-05-12

**Authors:** Weiping Sima, Meijuan Hu, Qiang He, Yixi Qiu, Yitao Lv, Lichun Dai, Qingwei Shao, Tao Zhou, Hong Li, Manyu Zhou, Hainan Ai, Hao Zhan

**Affiliations:** Department of Civil Engineering, Sichuan University of Science and Engineering Zigong 400045 P. R. China; Key Laboratory of Eco-Environment of Three Gorges Region, Ministry of Education, Chongqing University Chongqing 400044 P. R. China aihainan@126.com; Biomass Energy Technology Research Center, Biogas Institute of Ministry of Agriculture Chengdu 610041 P. R. China; The Tibet Autonomous Region Housing, Urban-rural Development Hall Job Training Center China; Chongqing Haoyang Water Construction Management Co., Ltd. Chongqing 400020 PR China

## Abstract

Correction for ‘Regulation of nitrogen dynamics at the sediment–water interface during HAB degradation and subsequent reoccurrence’ by Weiping Sima *et al.*, *RSC Adv.*, 2020, **10**, 13480–13488, DOI: 10.1039/c9ra10673a.

The authors regret that the affiliations of three of the authors (Hong Li, Hainan Ai and Hao Zhan) were shown incorrectly in the original article. The corrected author list and affiliations are as shown above.

The Royal Society of Chemistry apologises for these errors and any consequent inconvenience to authors and readers.

## Supplementary Material

